# Interpretable machine learning for identifying adolescent obesity risk and identifying key determinants

**DOI:** 10.3389/fpubh.2026.1657467

**Published:** 2026-02-25

**Authors:** Liepeng Huang, Jie Chen

**Affiliations:** 1 Faculty of Education, Shaanxi Normal University, Xi’an, Shaanxi, China; 2 Shandong Transport Vocational College, Weifang, Shandong, China

**Keywords:** adolescents, interpretable machine learning, obesity, relative importance, sedentary time

## Abstract

**Purpose:**

This study utilizes interpretable machine learning to identify and prioritize key associated factors for adolescent obesity across individual, family, and school domains, as well as to establish specific risk thresholds that can inform targeted interventions.

**Methods:**

Data were obtained from the China Education Panel Survey (CEPS), which included 7,397 adolescents. Six ML models (SVM, XGBoost, LightGBM, LR, RF, MLP) were developed and evaluated. The best-performing model was interpreted using SHAP analysis to assess feature contributions.

**Results:**

The LightGBM model demonstrated the highest accuracy (0.8788). This study primarily focused on the accurate classification of adolescent obesity status within a clinical decision-making context. Consequently, accuracy was prioritized as the key metric for directly assessing the model’s overall classification performance. Key predictors of this model sedentary time, school ranking, academic workload, birth weight, body image, family economic status, school location, household registration, and physical activity. Among these, sedentary behavior emerged as the most significant predictor. Specific risk thresholds were identified, including sedentary time exceeding 5 h on weekends and birth weight greater than 4.0 kg.

**Conclusion:**

This study underscores the utility of interpretable ML in identifying key predictors associated with adolescent obesity. The findings suggest that interventions might prioritize reducing sedentary behavior, the moderation of academic workload, and the enhancement of body image perception. Additionally, family and school environments play crucial roles in the prevention of obesity.

## 1 Introduction

In recent years, rapid economic development and significant improvements in living standards have rendered adolescent obesity a critical global public health challenge (1, 2). The global prevalence of adolescent obesity has doubled since 1990, becoming a critical public health challenge (3, 4). In China, the Report on Nutrition and Chronic Disease Status of Chinese Residents (5) indicates that the rates of overweight and obesity among children and adolescents aged 6 to 17 were 11.1 and 7.9%, respectively, in 2018, reflecting increases of 2.3 and 3.3% since 2012. Research evidence demonstrates that adolescent obesity is a significant risk factor for chronic diseases such as diabetes, hypertension, and hyperlipidemia (6–9), Furthermore, obesity during adolescence may persist into adulthood and later life (10), adversely affecting mental health and overall quality of life (11).

The early identification of high-risk groups and the implementation of targeted interventions can effectively prevent the onset and progression of adolescent obesity (12). However, the use of a logistic regression model for identifying obesity has limitations, primarily due to the limited number 6+.

6r of predictor variables and their inadequate predictive power, which hinders the ability to capture non-linear relationships among these variables (13, 14). To overcome this limitation, interpretable machine learning offers new avenues for analysis (15). Machine learning models are capable of capturing complex, non-linear relationships across a wide range of variables from various domains. Additionally, post-hoc interpretation tools, such as SHAP (Shapley Additive Explanations), can clarify model predictions and highlight actionable insights.

Therefore, this study identifies predictors of adolescent obesity by developing six machine learning models: Support Vector Machine (SVM), Extreme Gradient Boosting (XGBoost), Light Gradient Boosting Machine (LightGBM), Logistic Regression (LR), Random Forest (RF), and Multilayer Perceptron (MLP). Utilizing data from the China Education Panel Survey (CEPS) database, the study employs SHAP (Shapley Additive exPlanations) to interpret the prediction results of the best-performing models. This approach seeks to identify specific thresholds and the direction of associations among factors related to adolescent obesity, thereby examining the interrelationships among individual, family, and school domains associated with this condition. Ultimately, the findings are intended to provide actionable guidance for personalized health interventions. Existing machine learning studies on predicting adolescent obesity are limited in two significant ways. First, they typically concentrate on a single domain, neglecting to incorporate the synergistic effects of individual, family, and school factors. Second, even when interpretability tools are utilized, there is a persistent absence of actionable classification thresholds that can directly inform clinical or public health interventions across diverse populations. This study aims to address these gaps by developing a unified multi-domain model and identifying specific risk thresholds.

## 2 Data and methods

### 2.1 Data

#### 2.1.1 Data sources

The data utilized in this study originate from the China Education Panel Survey (CEPS), a longitudinal tracking survey developed and conducted by the China Survey and Data Centre at Renmin University of China (NSRC).^1^ This survey encompasses a diverse range of participants, including students, parents, teachers, and school leaders, and is among the first nationally representative longitudinal studies of secondary school students in China, emphasizing family, school, and community-level factors. All CEPS data received approval from the Institutional Review Board of the People’s University of China for research ethics concerning data collection within the CEPS dataset, and informed consent was obtained from all participants. Additionally, an ethical review for this study was conducted by Shandong Transport Vocational College.

#### 2.1.2 Data collection

##### 2.1.2.1 Obesity

The study focuses on the weight status of adolescents, utilizing the Body Mass Index (BMI) as the primary screening tool to monitor the effectiveness of anti-obesity campaigns (16). BMI is also recommended for clinical assessment of overweight and obesity in children and adolescents (17), calculated using the formula: body weight (kg)/height (m)^2^. According to China’s National Physical Fitness Standards for Students, a BMI of ≥ 25.3 indicates obesity in boys in the second year of junior high school, while a BMI of ≥ 24.9 indicates obesity in girls. Adolescents’ physical status is categorized into two groups: normal and obese. This study investigates the determinants associated with elevated BMI within an adolescent cohort. Anthropometric measurements, including height and weight, were obtained through self-report.

##### 2.1.2.2 Socio-demographic and behavioural characteristics

This study utilize data from the China Education Panel Survey (CEPS). Predictor variables were initially identified through a comprehensive literature review, with final selections made across individual, family, and school dimensions. Drawing from commonly used predictors in contemporary machine learning research on adolescent obesity, the following variables were included:

Individual-level variables:

Gender: Assessed by the question “What is your gender?”, coded as 0 = female, 1 = male.

Birth weight: Assessed by “What was your birth weight?”, categorized into low (<2,500 g), normal (2,500–4,000 g), and high (>4,000 g).

Household registration: Assessed by “What is your household registration type?”, coded as 0 = agricultural, 1 = non-agricultural.

Body image: Assessed by “How do you think you look?”, rated on a 5-point scale from 1 = very ugly to 5 = very beautiful.

Dietary habits: Assessed by “How often do you eat fried food, barbecue, snacks, or Western fast food?”, responses ranged from 1 = never to 5 = always.

Sleep quality: Evaluated using nine sleep-related issues (insomnia, easy awakening, drowsiness, no fatigue after waking, snoring, teeth grinding, dreaming, sleepwalking). The total number of reported issues was summed to form a continuous variable ranging from 0 to 9.

Sedentary time: Total hours spent on “watching TV” and “surfing the internet/gaming” during weekends; summed into a continuous variable (range: 0–12).

Academic workload: Time spent on academic activities during weekends; summed into a continuous variable (range: 3–18) based on three ordinal questions.

Weekend physical activity: Coded as 0 = no, 1 = yes.

Sports tutoring: “Do you attend sports tutoring classes?”, coded as 0 = no, 1 = yes.

Active commuting: “Do you use active commuting (walking or cycling)?”, coded as 0 = no, 1 = yes.

Physical activity frequency: Number of physical activity sessions per week, ranging from 0 to 7.

Family-level variables:

Paternal and maternal education: “What is your father’s/mother’s education level?”, coded as: 1 = no formal education, 2 = primary school, 3 = junior high school, 4 = vocational high school/technical school/regular high school, 5 = college, 6 = bachelor’s degree, 7 = master’s degree or above.

Family economic status: “What is your family’s current economic condition?”, rated on a 5-point scale from 1 = very difficult to 5 = very affluent.

Family structure: “Are you an only child?”, coded as 0 = no, 1 = yes.

School-level variables:

School type: Categorized as 1 = public school, 2 = private school, 3 = private school for migrant workers’ children.

School ranking: “What is your school’s current ranking in this district?”, rated from 1 = worst to 5 = best.

School location: Coded as 1 = rural, 2 = township, 3 = urban–rural fringe, 4 = suburban, 5 = central urban.

Sports facilities: Based on three items: “Does your school have a sports ground?”, “a gymnasium?”, and “a swimming pool?”; each coded as 1 = no, 2 = yes, and summed into a continuous score (range: 3–6).

#### 2.1.3 Data pre-processing

**Figure 1:**
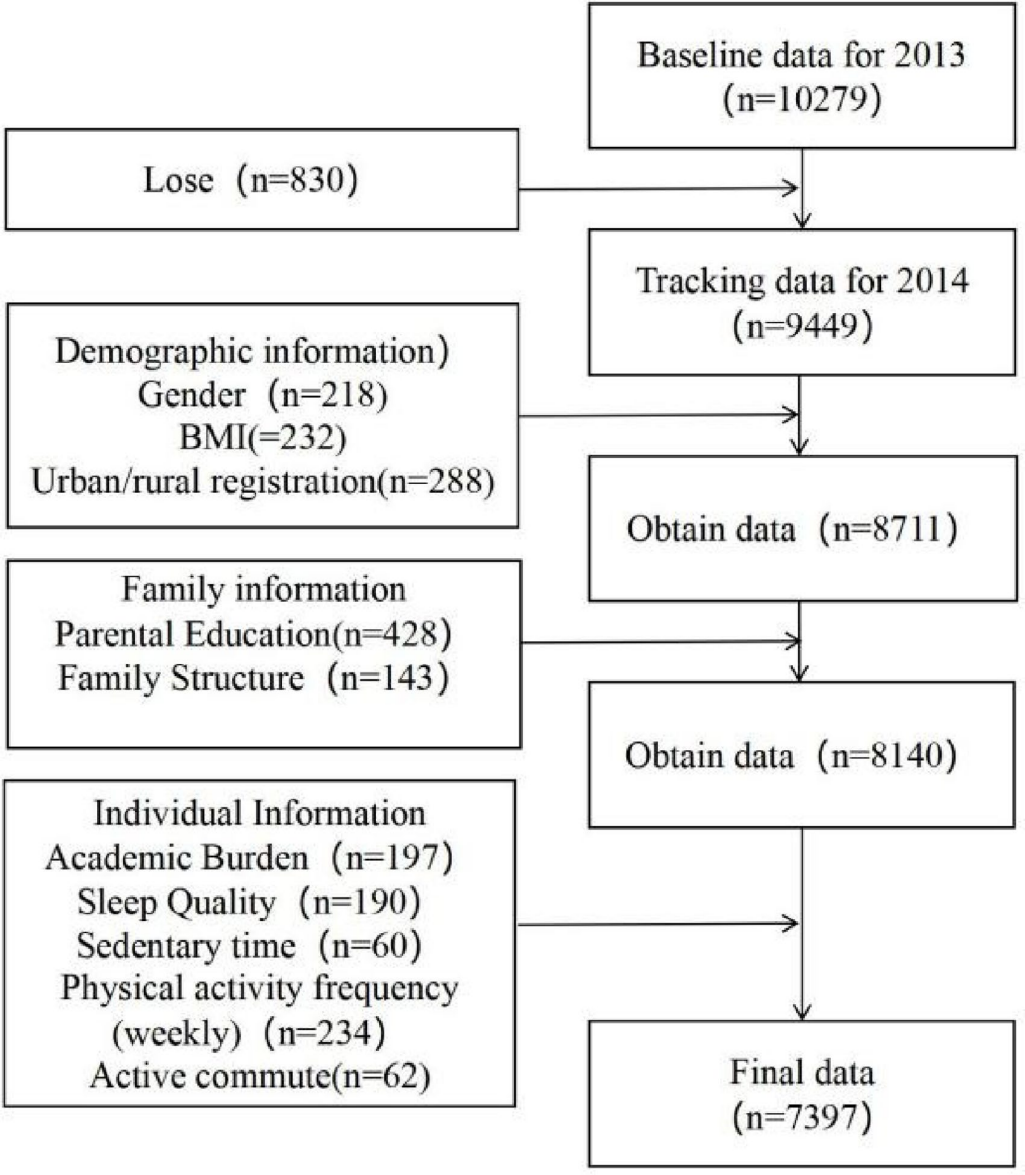
Participant selection flowchart.

This study employed a rigorous sample selection process for data processing and analysis. The initial pilot survey was conducted in 2012, followed by a nationwide survey during the 2013–2014 academic year. This nationwide survey included 28 county-level units across the country, from which 438 classes in 112 schools were randomly selected, involving a total of 10,279 seventh-grade students. By the follow-up survey in 2014, 9,449 participants were successfully tracked. Initial data cleaning and processing of the follow-up data resulted in 8,711 valid cases after the removal of invalid records. Subsequently, a statistical analysis of missing values was conducted for all variables. In accordance with methodological literature and principles of data integrity (18), the baseline 2013 data exhibited missing values across several variables, including demographic characteristics—gender (*n* = 218 missing), BMI (*n* = 232 missing), and urban–rural registration (*n* = 288 missing); family information—parental education (*n* = 428 missing) and family structure (*n* = 143 missing); and individual characteristics—academic burden (*n* = 197 missing), sleep quality (*n* = 190 missing), sedentary time (*n* = 60 missing), weekly physical activity frequency (*n* = 234 missing), and active commuting (*n* = 62 missing). Variables with missing rates below 5% were addressed using listwise deletion to preserve the structural integrity of the sample. For the key research variable “birth weight,” which exhibited a higher missing rate of 15.44%, a conditional imputation approach was implemented. The data were stratified by gender and household registration (agricultural vs. non-agricultural), with missing birth weight values replaced by the mode within each subgroup. This methodology maximized sample retention while maintaining group representativeness, thereby ensuring scientifically sound and reasonable data imputation (19–21). The final analytical sample consisted of 7,397 participants (see Figure 1).

### 2.2 Research methodology

#### 2.2.1 Feature screening methods

**Figure 2:**
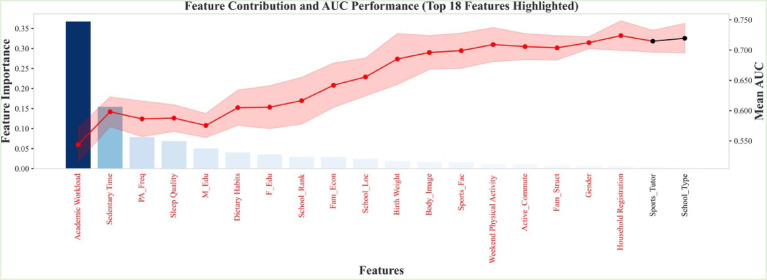
Recursive feature screening based on random forests (top 18).

The dataset comprised a total of 7,397 participants, of whom 6,356 were classified into the normal weight group and 1,041 into the obese group. To mitigate overfitting and evaluate the generalization capability of the predictive models, the sample was randomly partitioned into a training set (70%) and a test set (30%) (22). All data preprocessing and model development were conducted exclusively on the training set. Subsequently, 18 predictor variables were subjected to feature selection using Recursive Feature Elimination (RFE) (see Figure 2). Based on the refined feature subset, six machine learning algorithms were employed to construct risk prediction models for adolescent obesity: Support Vector Machine (SVM), Extreme Gradient Boosting (XGBoost), Light Gradient Boosting Machine (LightGBM), Logistic Regression (LR), Random Forest (RF), and Multilayer Perceptron (MLP). These algorithms have been extensively utilized in prior research concerning obesity prediction in pediatric and adolescent populations (23, 24).

#### 2.2.2 Machine learning methods

Utilizing Python’s sci-kit-learn library and the LightGBM library, six machine-learning models were developed based on 18 selected feature variables. The algorithms employed were: Support Vector Machine (SVM), Extreme Gradient Boosting (XGBoost), Light Gradient Boosting Machine (LightGBM), Logistic Regression (LR), Random Forest (RF), and Multilayer Perceptron (MLP). The performance of these six machine learning models was compared across various characteristics. Logistic Regression (LR) provides a computationally efficient and interpretable baseline (25); Support Vector Machine (SVM) excels in high-dimensional spaces and nonlinear classification; XGBoost delivers high-accuracy prediction on structured data (26); Gradient Boosting Machine (LightGBM) can flexibly fit complex patterns (27); RF is robust and facilitates feature significance analysis (28), while Multi-Layer Perceptron (MLP) can approximate arbitrary complex functions (29). The optimal hyperparameters for each model were determined through Grid Search, aiming to minimize overfitting and enhance predictive performance. The optimal parameter configurations for each model are as follows: LR (C: 1, penalty: l2, solver: lbfgs); RF (max_depth: 10, min_samples_split: 5, n_estimators: 200); SVM (C: 1, gamma: auto, kernel: rbf); XGBoost (learning_rate: 0.1, max_depth: 3, n_estimators: 100); LightGBM (boosting_type: dart, learning_rate: 0.1, n_estimators: 200, num_leaves: 31); MLP (hidden_layer_sizes: 100, learning_rate_init: 0.01, max_iter: 200). The optimal parameter configurations obtained through Grid Search effectively mitigate the risk of overfitting and significantly enhance the predictive performance of each machine learning model on the dataset.

#### 2.2.3 Model evaluation and metric selection

**Figure 3:**
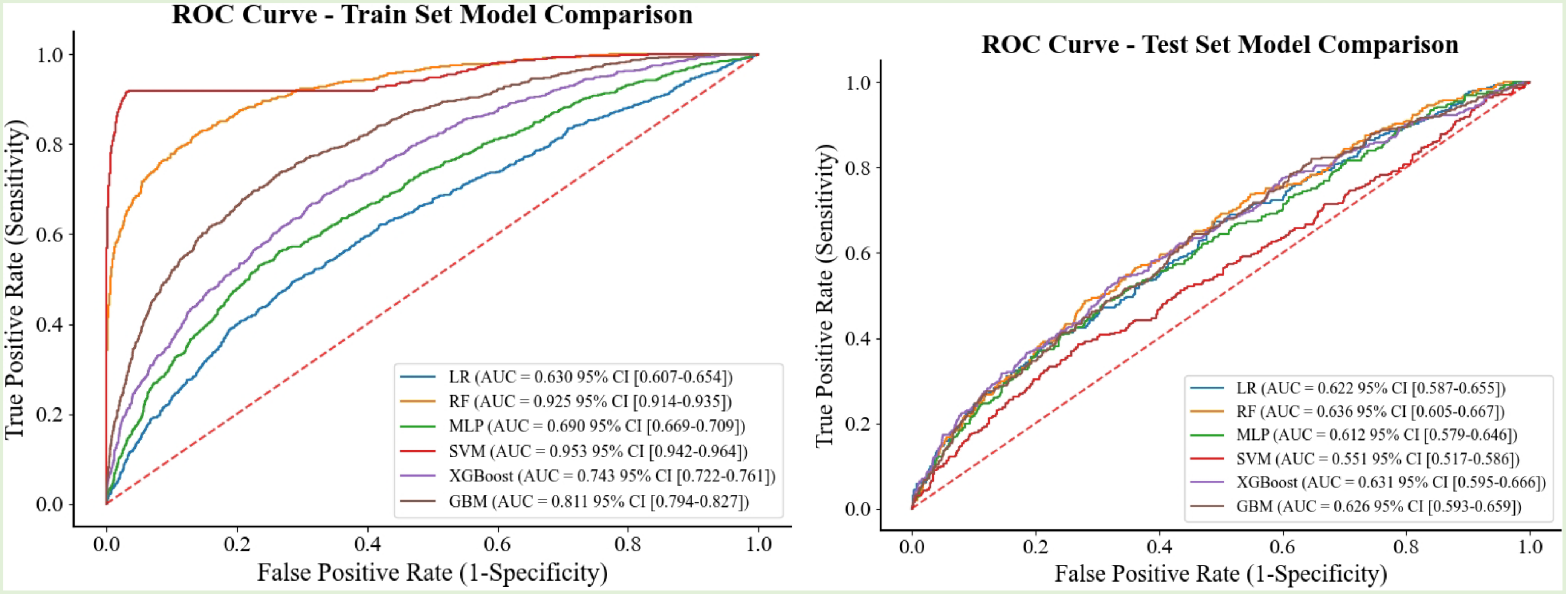
Comparison of ROC curves between training and test sets.

The primary clinical decision-making scenario of this study centered on the accurate classification of obesity status rather than on ranking risk probabilities. Consequently, accuracy was chosen as the core optimization metric to directly reflect the model’s overall capacity for making correct classifications. While the area under the curve (AUC) is a robust metric for evaluating a model’s discriminative ability, accuracy offers a more intuitive and actionable performance measure for our specific application goals (see Figure 3). For a comprehensive comparison and reference, precision, sensitivity, and AUC were also calculated and reported alongside accuracy (see Supplementary material 1 for details).

#### 2.2.4 SHAP interpretable analysis methods

Shapley’s explanatory approach, grounded in game theory, offers a robust framework for ensuring consistency, local fidelity and addressing missingness in any machine learning model (30). Central to this approach is the capacity to evaluate the contribution of all predictor variables to model outcomes by converting the predictions into marginal contribution values for each variable (18, 30). This methodology facilitates both individualized interpretations and broader insights into adolescent obesity prediction models. Higher SHAP values signify a positive influence of a variable on the model output, while lower values indicate a negative influence (19).

#### 2.2.5 Methods of statistical analysis

Continuous variables were analyzed and compared according to the results of the normality test (Shapiro–Wilk method). Variables exhibiting a normal distribution were reported as mean ± standard deviation, with group comparisons conducted using the independent samples *t*-test. In contrast, variables with a non-normal distribution were presented as median (interquartile range), and group comparisons were performed using the Mann–Whitney *U* test. Categorical variables were expressed as frequencies (percentages), with group comparisons based on the appropriate statistical test: the Pearson chi-square test was applied when the minimum expected frequency exceeded 5, while Fisher’s exact test was utilized when it was 5 or fewer. All statistical analyses were conducted using two-sided tests, with a significant level set at *p* < 0.05.

## 3 Results

### 3.1 Participant characteristics

The study included 7,397 participants, categorized by weight status into a normal weight group (*n* = 6,356, 85.9%) and an obese group (*n* = 1,041, 14.1%). The rate of adolescent obesity is largely consistent with findings from previous studies (20, 21, 31, 32). Differences across 20 variables between the two groups were assessed using chi-square tests (*χ*^2^) (see Supplementary material 2 for details).

Individual-level factors: Statistically significant differences (*p* < 0.05) were observed between the groups in gender, birth weight, urban/rural domicile, body image, dietary habits, sedentary time, and frequency of physical activity. Specifically, the obese group contained a higher proportion of males, more urban residents, individuals with heavier birth weight, lower body image perception, less healthy dietary habits, longer sedentary time, and lower frequency of physical activity.

Family-level factors: Significant differences (*p* < 0.05) were identified in family economic status and family structure. Adolescents from very wealthy families had an obesity rate of 7.20%, showing a “U-shaped” distribution across economic levels. Additionally, the obesity rate among only-child families was 55.04%.

School-level factors: Significant differences (*p* < 0.05) were found in school ranking and school location between the obese and normal weight groups. Furthermore, weekend sports participation, attendance in sports counseling, and sleep quality were significantly lower in the obese group.

### 3.2 Predictive performance of machine learning models

Six machine learning models were used to predict the incidence of obesity in adolescents. The performance of these models is shown in Table 1 and illustrated mainly by ROC and Accuracy. A side-by-side comparison of SVM, XGBoost, LightGBM, LR, RF, and MLP in the training set and the test model reveals that the LightGBM model exhibits the best discriminative ability (accuracy = 0.8788, AUC = 0.7392), with a significant advantage over the other models (33).

**Table 1:** Six machine learning prediction model scores.

Model	Dataset	Accuracy	AUC
SVM	Train	0.8856	0.8911
SVM	Test	0.8658	0.6765
XGBoost	Train	0.8864	0.8108
XGBoost	Test	0.8779	0.7450
LightGBM	Train	0.8997	0.9062
LightGBM	Test	0.8788	0.7392
LR	Train	0.8685	0.7168
LR	Test	0.8712	0.7194
R F	Train	0.8967	0.8999
R F	Test	0.8770	0.739
MLP	Train	0.8723	0.7625
MLP	Test	0.8725	0.7143

### 3.3 Decision curve analysis

To assess the clinical utility of the predictive model in facilitating decision-making, we employed Decision Curve Analysis (DCA) for validation (34). The results of the DCA for both the training and test sets are presented in Supplementary material 3. The DCA results for the test set demonstrated that the machine learning model possesses clinical utility. This study established a threshold probability range of 10–60% as reasonable for clinical decision-making. This range is consistent with the conventions of decision curve analysis in the field of childhood obesity risk prediction (35), indicating that utilizing our Gradient Boosting Machine (LightGBM) model to inform decisions would yield superior outcomes, exhibiting a higher net benefit compared to the strategies of “intervene for all” and “intervene for none.” The performance of each model in the training set exhibited a similar, albeit more optimistic, trend, which aligns with our expectations.

### 3.4 Relative importance of combined factors on adolescent obesity

**Figure 4:**
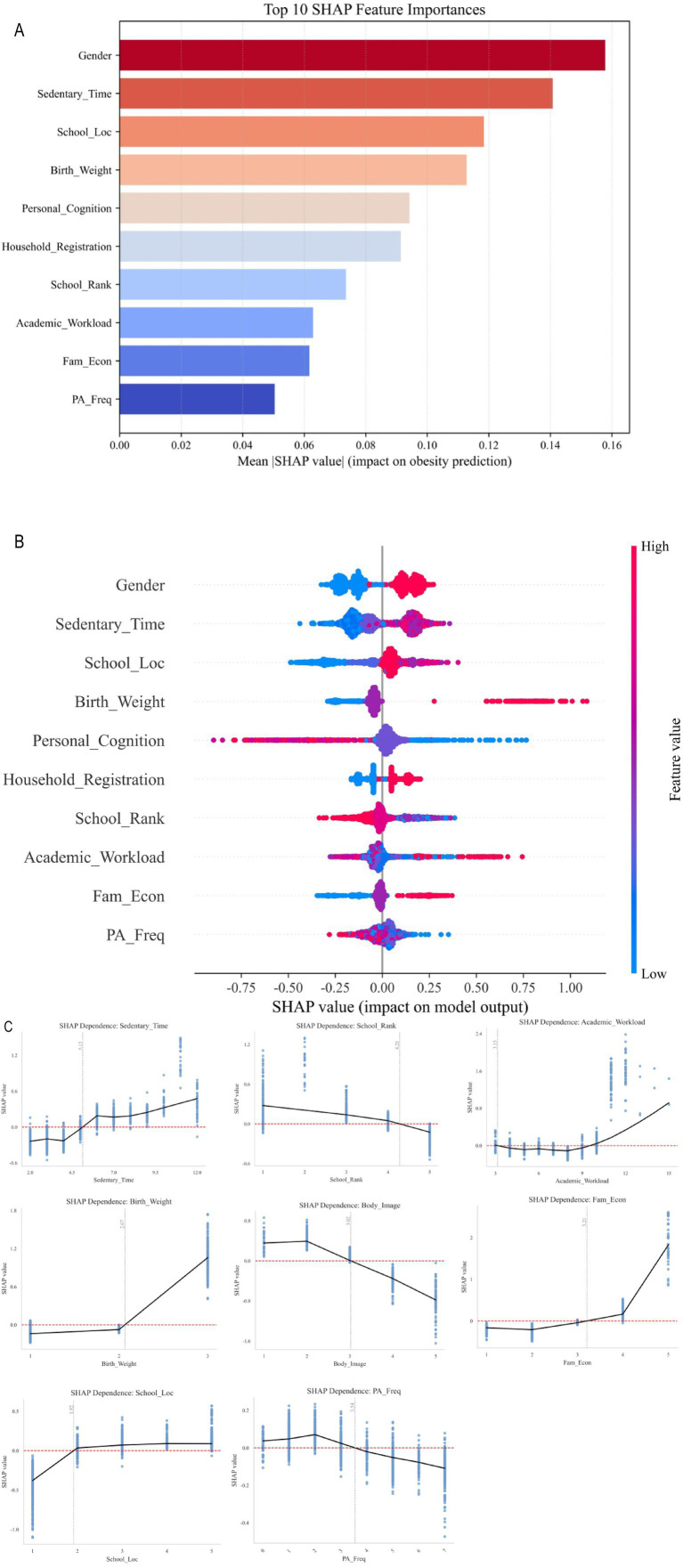
Relative importance of combined factors on adolescent obesity. **(A)** Feature importance; **(B)** SHAP results; **(C)** Impact of features on model output, from top left to bottom right: Sedentary time, school ranking, academic workload, birth weight, body image, family economy, school location, household registration, and physical activity. The horizontal axis represents the actual value of a particular feature, while the vertical axis represents the corresponding SHAP value for that feature (i.e., the effect of the characteristic on the model output: positive values indicate a positive effect, while negative values indicate a negative effect), PA, physical activity.

The Gradient Boosting Machine (LightGBM) demonstrated superior performance in explaining adolescent obesity. Figure 4A illustrates the ranking of variable importance within the model, specifically highlighting the 10 characteristics that exert the most significant influence on adolescent obesity. Among these, sedentary time is identified as the most critical risk factor. The X-axis indicates that Higher SHAP values indicate a stronger positive association with the model’s prediction of obesity, corresponding to an increased predicted likelihood of obesity, while lower values suggest a reduced likelihood of obesity. A color gradient ranging from red to blue represents the magnitude of feature values, with red denoting high values and blue indicating low values. Furthermore, an increased feature value associated with school location preference (e.g., central city), high academic workload, high birth weight, low self-assessment of body image, favorable family economic conditions (middle class and above), non-agricultural domicile, low school ranking, and infrequent physical activity were also recognized as significant predictors of adolescent obesity (Figure 4B).

Specifically, adolescents who engage in more than 5 h of sedentary activity per day on weekends, participate in fewer than 3.5 physical activity sessions per week, and possess an academic workload value of less than 3 or greater than 10 are at an elevated risk of obesity. Additionally, school rank is negatively correlated with the risk of obesity in adolescents, while those with birth weights exceeding the normal range (>4.0 kg) also face an increased risk. Furthermore, adolescents who have a low self-assessment of body image (≤3) are at heightened risk of obesity. In contrast, adolescents from families with medium to high economic status and those attending schools located above the township level are predicted to be at an increased risk of obesity.

## 4 Discussion

In alignment with the complex and multifactorial nature of adolescent obesity, this study utilized an interpretable machine learning approach to assess the relative significance of individual, family, and school factors in identifying obesity among adolescents. Utilizing data from the China Education Panel Survey (CEPS) comprising 7,397 valid samples, we identified several predisposing factors across these three dimensions. Notably, sedentary time emerged as the strongest predictor, consistent with previous studies that underscore its central role in obesity prevention (36–38). This study is the first to integrate individual, family, and school factors within a unified model, offering a more comprehensive perspective on the combined influences affecting adolescent obesity.

At the family level, economic status is a significant determinant of obesity. Adolescents from medium- and high-income families exhibit a higher likelihood of obesity, consistent with existing literature (39, 40). Improved economic conditions may be associated with over-nutrition (41, 42), characterized by increased consumption of high-sugar beverages and fast food due to greater disposable income and allowances (43). Additionally, these conditions may correlate with reduced physical activity facilitated by motorized transportation (44, 45). Collectively, these potential pathways may explain the observed association with an elevated risk of obesity. Therefore, fostering healthy family environments and behavioral patterns is essential to avoid the “convenience trap” and promote active lifestyles and balanced diets.

Another notable finding is the elevated obesity rate among adolescents from only-child families (55.04%). This finding aligns with the results of Formisano (46), Hunsberger (47), and others. This phenomenon may be attributed to concentrated family resources leading to over-nutrition, heightened academic expectations resulting in reduced physical activity (48), increased stress-related eating (49), or differing parenting styles (50). This underscores the necessity for targeted nutritional and lifestyle guidance within single-child family contexts.

School-related factors, such as proximity to urban centers and lower school rankings, are associated with higher an increased risk of obesity. Schools in central urban areas often lack safe infrastructure for walking and cycling (51), and they are typically surrounded by greater access to high-calorie foods (52, 53). Additionally, Lower-ranked schools may provide inadequate physical education resources, subpar curriculum quality, and insufficient nutritional offerings (54, 55). In contrast, higher-ranked schools tend to prioritize holistic student development, including physical activity and healthy eating (56, 57). These findings highlight the necessity of enhancing the physical environments of schools, strengthening physical and health education programs, and ensuring nutritional balance in school meals.

At the individual level, gender, sedentary time, birth weight, body image, academic workload, and frequency of physical activity emerged as significant predictors. Both intrinsic factors (e.g., gender, birth weight, household registration) and extrinsic factors (e.g., sedentary behavior, body image, academic load, and physical activity) exerted considerable influence.

Birth weight: Normal newborns typically have a birth weight ranging from 2,500 to 4,000 grams (58). This study indicates that exceeding the normal birth weight is a significant predictor of obesity in adolescents. These findings are consistent with two systematic reviews that examined the relationship between birth weight and obesity in children and adolescents, underscoring the strong association between high birth weight and obesity, as well as the protective effect of low birth weight (59, 60). Children born with high birth weight often have a greater number of adipocytes and an increased capacity for fat storage, rendering them susceptible to fat accumulation when excess energy is available (61). Furthermore, this condition is associated with maternal weight and dietary habits both before and after pregnancy (62, 63), as well as with overfeeding behaviors in the home (64). Therefore, the long-term importance of maternal health management and evidence-based infant and young child feeding practices cannot be overstated.

Body image: This study demonstrated an association between low self-assessment of body image and an increased risk of obesity, consistent with findings from an Australian study (65). Evidence indicates that a higher body mass index (BMI) is consistently linked to poor body image and body dissatisfaction (66). Students who possess high body image satisfaction or acceptance exhibit greater self-efficacy and self-esteem (67) and are more likely to engage in and maintain healthy lifestyle behaviors (68), which could be a pathway linking body image to obesity risk. While simultaneously mitigating negative emotions such as anxiety, depression, and stress (69), which can directly or indirectly promote unhealthy behaviors (70) that increase energy intake and the risk of obesity. Additionally, the aesthetic notion that “thinness is beautiful” (71, 72) may motivate adolescents to manage their body weight, thereby reducing their risk of obesity. Consequently, obesity prevention efforts targeting adolescents should also emphasize mental health and the cultivation of accurate and positive body awareness, as well as intrinsic health motivation.

This study demonstrates an association between low self-assessment of body image and an increased risk of obesity, consistent with findings from an Australian study (65). Evidence indicates that a higher body mass index (BMI) is consistently linked to poor body image and body dissatisfaction (66). Students who exhibit high body image satisfaction or acceptance demonstrate greater self-efficacy and self-esteem (67) and are more likely to engage in and maintain healthy lifestyle behaviors (68). This relationship may serve as a pathway linking body image to obesity risk while simultaneously mitigating negative emotions such as anxiety, depression, and stress (69), which can directly or indirectly promote unhealthy behaviors (70) that increase energy intake and the risk of obesity. Furthermore, the aesthetic notion that “thinness is beautiful” (71, 72) may motivate adolescents to manage their body weight, thereby reducing their risk of obesity. Consequently, obesity prevention efforts targeting adolescents should also emphasize mental health and the cultivation of accurate and positive body awareness, as well as intrinsic health motivation.

Sedentary time refers to prolonged periods of sedentary behavior, such as watching television, playing video games, or using a computer (73). This study indicates that longer sedentary time is associated with a higher risk of obesity in adolescents, a finding that aligns with existing academic research (74, 75). Sedentary behavior is generally associated with decreased levels of physical activity and daily energy expenditure, which in turn leads to a diminished rate of fat oxidation in the muscles (76). When energy expenditure is lower than energy intake, the excess energy is converted into fat for storage (77). Furthermore, sedentary behavior represents an allocation of time resources in adolescents’ daily lives, leading to decreased physical activity and disrupted rest due to the crowding out of active pursuits. Consequently, this contributes to the development of obesity.

Academic workload: Our analysis revealed a U-shaped association between academic workload and obesity risk, indicating that values below 3 or above 10 significantly elevate this risk. Existing studies have demonstrated that a high academic workload is associated with obesity in adolescents (78); this is primarily due to excessive academic pressure, which extends study hours, reduces physical activity, and disrupts sleep quality (79). Concurrently, such pressure may heighten adolescents’ cravings for high-sugar and high-fat foods (80), contributing to fat accumulation and an increased likelihood of obesity. Additionally, our study found that a low academic workload also correlates with an increased risk of obesity. However, there is a relative scarcity of research exploring the positive association between low academic workload and obesity. This may be attributed to poor time management, which can lead to increased sedentary behavior and irregularities in lifestyle that adversely affect diet and physical activity. Further research is necessary to validate the underlying mechanisms and prevalence of these associations.

Physical Activity Frequency: This study demonstrates that the frequency of physical activity is negatively associated with obesity in adolescents. Previous research indicates that participation in physical activity is the most effective strategy for preventing obesity in this population (81). Physical activity induces beneficial changes in various health metrics, including fat percentage, waist circumference, systolic blood pressure, insulin levels, LDL cholesterol, and total cholesterol (82). Additionally, it enhances basal energy metabolism, improves body composition, and promotes healthy sleep and psychological well-being. The multifactorial effects of these changes can significantly mitigate the onset and progression of obesity among adolescents.

It is crucial to further delineate the modifiable and non-modifiable associated factors identified in this study. Factors such as birth weight and school location are not easily altered through individual behavior; however, their strong association with the risk of adolescent obesity renders them valuable screening indicators for the early identification of high-risk individuals. Consequently, adolescents with a high birth weight (greater than 4.0 kg) or those attending schools in central urban areas should be prioritized for enhanced screening and targeted lifestyle interventions that focus on modifiable factors, such as sedentary behavior and physical activity levels. This approach aligns with precision public health strategies, facilitating a more efficient allocation of resources to subgroups that are most in need of intervention.

This study developed six machine learning models—Support Vector Machine (SVM), XGBoost, Gradient Boosting Machine (LightGBM), Logistic Regression (LR), Random Forest (RF), and Multi-Layer Perceptron (MLP)—to predict adolescent obesity. The area under the curve (AUC) value of 0.7392 reported in this study is lower than the range of 0.84 to 0.91 found in Li et al.’s study on adults (85). This discrepancy may arise from several factors. First, the study populations differ, with adolescents exhibiting more complex and multifaceted influencing factors for obesity compared to adults. Second, this study included a broader range of social environmental and school-level characteristics, which may demonstrate stronger non-linear relationships with the outcome and introduce additional noise (86). Finally, the use of self-reported height and weight to calculate body mass index (BMI) may have introduced measurement error. Although the AUC is moderate, the high classification accuracy of 0.8788 is of greater practical significance for effectively classifying individuals within the population, particularly in the context of large-scale screening. In conclusion, we contend that an AUC of 0.7392 is acceptable within the framework of this study.

Finally, this study identified actionable thresholds: >5 h of sedentary time on weekends, <3.5 physical activity sessions per week, academic workload <3 or >10, birth weight >4.0 kg, body image score ≤3, and medium/high family economic status were associated with increased obesity risk. These align with the Technical Guidelines for Comprehensive Public Health Prevention and Control of Overweight and Obesity in Primary and Secondary School Students (2024), which recommend 3–4 moderate-to-vigorous physical activity sessions per week, limited sedentary and screen time, and emphasize the roles of family and school in obesity prevention.

Several limitations must be acknowledged. Identifying factors associated with adolescent obesity across individual, family, and school domains is complex due to the multitude of potential influences; consequently, certain relevant factors may not have been included in the current study’s model. This study employed a cross-sectional design, which, while revealing associations between variables, cannot infer causality. Furthermore, key indicators such as height and weight were based on self-reports. Although several studies have indicated moderate to high correlations between self-reported and measured anthropometric data in adolescent populations (83, 84), measurement bias is inevitable and may lead to the underestimation or overestimation of obesity prevalence. Future research should adopt longitudinal designs and incorporate objective measurements to validate and extend these findings. Additionally, including objective data, such as biochemical indicators, would enhance the richness of the sample. Regarding the feature selection strategy, although Recursive Feature Elimination with cross-validation was effectively employed to prevent overfitting and information leakage, we did not systematically compare it with alternative strategies (e.g., Lasso regularization path, filter methods based on mutual information, or embedded methods). Consequently, the selected feature subset represents one of many potentially effective combinations and may not be globally optimal. Future studies could compare multiple feature selection strategies to assess the stability of both the final model performance and the selected features. Finally, although SHAP provides valuable insights, it remains a post-hoc interpretation tool and does not fully address the “black-box” nature of complex models like Gradient Boosting Machines (LightGBM). Therefore, it is essential to explore the use of more sophisticated and accurate prediction models to improve predictive performance and continuously enhance the interpretability of the results.

## 5 Conclusion

The data for this study were obtained from the China Education Tracking Survey (CEPS) database, which included 7,397 adolescents. Six machine learning models were developed to predict the risk of adolescent obesity using the CEPS database, with the Gradient Boosting Machine (LightGBM) model demonstrating superior overall predictive accuracy and stability compared to the other five models. The study revealed demographic differences in adolescent obesity and underscored the relative importance of factors such as sedentary time, school zone, birth weight, and body image. This research proposes three evidence-based intervention targets: (1) limiting recreational screen time to less than 2 h per day, (2) ensuring a moderate academic workload (3–10 on a standardized scale), and (3) enhancing body image literacy. These targets aim to inform the development of interventions for preventing and managing adolescent obesity. The specific risk thresholds identified for certain factors in this study may guide future measures for adolescent obesity prevention. Furthermore, additional individual, school, and family factors should be incorporated into future research to assess the generalizability of these findings.

## Data Availability

The datasets presented in this study can be found in online repositories. The names of the repository/repositories and accession number(s) can be found in the article/Supplementary material.

## Footnotes

1
http://ceps.ruc.edu.cn

## References

[1] James WPT. Obesity: a global public health challenge. Clin Chem. (2018) 64:24–9. doi: 10.1373/clinchem.2017.273052, 29295834

[2] Edward S Gopalakrishnan S. Adolescent obesity–emerging public health problem of 21st century. Natl J Community Med. (2022) 13:43–8. doi: 10.5455/njcm.20211020091723

[3] Okunogbe A Nugent R Spencer G Powis J Ralston J Wilding J. Economic impacts of overweight and obesity: current and future estimates for 161 countries. BMJ Glob Health. (2022) 7:e009773. doi: 10.1136/bmjgh-2022-009773, 36130777 PMC9494015

[4] Ge C Xiong J Zhu R Hong Z He Y. The global burden of high BMI among adolescents between 1990 and 2021. Commun Med. (2025) 5:125. doi: 10.1038/s43856-025-00838-2, 40247108 PMC12006325

[5] The National Health and Family Planning Commission. Report on nutrition and chronic disease status of Chinese residents. Beijing: People’s Medical Publishing House (2020).

[6] Lobstein T Baur L Uauy R. Obesity in children and young people: a crisis in public health. Obes Rev. (2004) 5 Suppl 1:4–104. doi: 10.1111/j.1467-789X.2004.00133.x, 15096099

[7] Wang YC Mcpherson K Marsh T Gortmaker SL Brown M. Health and economic burden of the projected obesity trends in the USA and the UK. Lancet. (2011) 378:815–25. doi: 10.1016/s0140-6736(11)60814-3, 21872750

[8] Puhl RM Heuer CA. The stigma of obesity: a review and update. Obesity. (2009) 17:941–64. doi: 10.1038/oby.2008.636, 19165161

[9] Stunkard AJ Faith MS Allison KC. Depression and obesity. Biol Psychiatry. (2003) 54:330–7. doi: 10.1016/s0006-3223(03)00608-5, 12893108

[10] Bixby H Mishra A Martinez A. R. (2025). Worldwide levels and trends in childhood obesity. In: Childhood obesity. Elsevier: Academic Press. 21–40.

[11] Swallen KC Reither EN Haas SA Meier AM. Overweight, obesity, and health-related quality of life among adolescents: the National Longitudinal Study of adolescent health. Pediatrics. (2005) 115:340–7. doi: 10.1542/peds.2004-0678, 15687442

[12] Barlow SE Committee E. Expert committee recommendations regarding the prevention, assessment, and treatment of child and adolescent overweight and obesity: summary report. Pediatrics. (2007) 120:S164–92. doi: 10.1542/peds.2007-2329C 18055651

[13] Marcos-Pasero H Colmenarejo G Aguilar-Aguilar E Ramírez de Molina A Reglero G Loria-Kohen V. Ranking of a wide multidomain set of predictor variables of children obesity by machine learning variable importance techniques. Sci Rep. (2021) 11:1910. doi: 10.1038/s41598-021-81205-8, 33479310 PMC7820584

[14] Zhang S Tjortjis C Zeng X Qiao H Buchan I Keane J. Comparing data mining methods with logistic regression in childhood obesity prediction. Inf Syst Front. (2009) 11:449–60. doi: 10.1007/s10796-009-9157-0

[15] Colmenarejo G. Machine learning models to predict childhood and adolescent obesity: a review. Nutrients. (2020) 12:2466. doi: 10.3390/nu12082466, 32824342 PMC7469049

[16] Khanna D Peltzer C Kahar P Parmar MS. Body mass index (BMI): a screening tool analysis. Cureus. (2022) 14:e22119. doi: 10.7759/cureus.22119 35308730 PMC8920809

[17] Daniels SR. The use of BMI in the clinical setting. Pediatrics. (2009) 124:S35–41. doi: 10.1542/peds.2008-3586F 19720666

[18] Giudici P Raffinetti E. Shapley-Lorenz eXplainable artificial intelligence. Expert Syst Appl. (2021) 167:114104. doi: 10.1016/j.eswa.2020.114104

[19] Wang H Liang Q Hancock JT Khoshgoftaar TM. Feature selection strategies: a comparative analysis of SHAP-value and importance-based methods. J Big Data. (2024) 11:44. doi: 10.1186/s40537-024-00905-w

[20] Zhen S Ma Y Zhao Z Yang X Wen D. Dietary pattern is associated with obesity in Chinese children and adolescents: data from China health and nutrition survey (CHNS). Nutr J. (2018) 17:68. doi: 10.1186/s12937-018-0372-8, 29996840 PMC6042200

[21] Zhu Z Yin P. Overweight and obesity: the serious challenge faced by Chinese children and adolescents. J Glob Health. (2023) 13:03036. doi: 10.7189/jogh.13.03036, 37469286 PMC10357130

[22] Xu Y Goodacre R. On splitting training and validation set: a comparative study of cross-validation, bootstrap and systematic sampling for estimating the generalization performance of supervised learning. J Anal Test. (2018) 2:249–62. doi: 10.1007/s41664-018-0068-2, 30842888 PMC6373628

[23] Gupta M Phan T-LT Bunnell HT Beheshti R. Obesity prediction with EHR data: a deep learning approach with interpretable elements. ACM Trans Comput Healthcare. (2022) 3:1–19. doi: 10.1145/3506719, 35756858 PMC9221869

[24] Lee S Chun J. Identification of important features in overweight and obesity among Korean adolescents using machine learning. Child Youth Serv Rev. (2024) 161:107644. doi: 10.1016/j.childyouth.2024.107644

[25] Rudin C. Stop explaining black box machine learning models for high stakes decisions and use interpretable models instead. Nat Mach Intell. (2019) 1:206–15. doi: 10.1038/s42256-019-0048-x, 35603010 PMC9122117

[26] Ester M Kriegel H Xu X. Xgboost: a scalable tree boosting system. In Proceedings of the 22nd ACM SIGKDD International Conference on Knowledge Discovery and Data Mining (785, 2016). (2022).

[27] Ke G Meng Q Finley T . LightGBM: a highly efficient gradient boosting decision tree. Adv Neural Inf Proces Syst. (2017) 30

[28] Breiman L. Random forests. Mach Learn. (2001) 45:5–32. doi: 10.1023/a:1010933404324

[29] Hornik K Stinchcombe M White H. Multilayer feedforward networks are universal approximators. Neural Netw. (1989) 2:359–66. doi: 10.1016/0893-6080(89)90020-8

[30] Lundberg SM Erion G Chen H DeGrave A Prutkin JM Nair B . From local explanations to global understanding with explainable Ai for trees. Nat Mach Intell. (2020) 2:56–67. doi: 10.1038/s42256-019-0138-9, 32607472 PMC7326367

[31] Guo Y Yin X Wu H Chai X Yang X. Trends in overweight and obesity among children and adolescents in China from 1991 to 2015: a meta-analysis. Int J Environ Res Public Health. (2019) 16:4656. doi: 10.3390/ijerph16234656, 31766709 PMC6926698

[32] Hong Y Ullah R Wang J-B Fu JF. Trends of obesity and overweight among children and adolescents in China. World J Pediatr. (2023) 19:1115–26. doi: 10.1007/s12519-023-00709-7, 36920656 PMC10015139

[33] Yacouby R Axman D. Probabilistic extension of precision, recall, and F1 score for more thorough evaluation of classification models; In Proceedings of the First Workshop on Evaluation and Comparison of NLP Systems, (2020).

[34] Vickers AJ Holland F. Decision curve analysis to evaluate the clinical benefit of prediction models. Spine J. (2021) 21:1643–8. doi: 10.1016/j.spinee.2021.02.024, 33676020 PMC8413398

[35] Zheng M Zhang Y Laws RA Vuillermin P Dodd J Wen LM . Development of machine learning-based risk prediction models to predict rapid weight gain in infants: analysis of seven cohorts. JMIR Public Health Surveill. (2025) 11:e69220. doi: 10.2196/69220 40532141 PMC12192193

[36] Liou YM Liou TH Chang LC. Obesity among adolescents: sedentary leisure time and sleeping as determinants. J Adv Nurs. (2010) 66:1246–56. doi: 10.1111/j.1365-2648.2010.05293.x, 20546358

[37] Baur LA. Child and adolescent obesity in the 21st century: an Australian perspective. Asia Pac J Clin Nutr. (2002) 11 Suppl 3:S524–8. doi: 10.1046/j.1440-6047.11.supp3.9.x, 12492643

[38] Thibault H Contrand B Saubusse E Baine M Maurice-Tison S. Associated factors for overweight and obesity in French adolescents: physical activity, sedentary behavior and parental characteristics. Nutrition. (2010) 26:192–200. doi: 10.1016/j.nut.2009.03.015, 19577429

[39] Fernandes RA Christofaro DGD Cardoso JR Ronque ERV Freitas Júnior IF Kawaguti SS . Socioeconomic status as determinant of associated factors for overweight in adolescents. Ciencia Saude Coletiva. (2011) 16:4051–7. doi: 10.1590/S1413-81232011001100010, 22031134

[40] Gallego A López-Gil JF. The role of individual and contextual economic factors in obesity among adolescents: a cross-sectional study including 143 160 participants from 41 countries. J Glob Health. (2024) 14:04035. doi: 10.7189/jogh.14.04035, 38389438 PMC10884718

[41] Schneider D. International trends in adolescent nutrition. Soc Sci Med. (2000) 51:955–67. doi: 10.1016/s0277-9536(00)00074-5, 10972438

[42] Ruiz LD Zuelch ML Dimitratos SM Scherr RE. Adolescent obesity: diet quality, psychosocial health, and cardiometabolic associated factors. Nutrients. (2019) 12:43. doi: 10.3390/nu12010043 31877943 PMC7020092

[43] Gan WY Mohamed SF Law LS. Unhealthy lifestyle associated with higher intake of sugar-sweetened beverages among Malaysian school-aged adolescents. Int J Environ Res Public Health. (2019) 16:2785. doi: 10.3390/ijerph16152785, 31382672 PMC6696103

[44] Gao Y Chen X Li T Chen F. Differences in pupils’ school commute characteristics and mode choice based on the household registration system in China. Case Stud Transp Policy. (2017) 5:656–61. doi: 10.1016/j.cstp.2017.07.008

[45] Faulkner GE Buliung RN Flora PK Fusco C. Active school transport, physical activity levels and body weight of children and youth: a systematic review. Prev Med. (2009) 48:3–8. doi: 10.1016/j.ypmed.2008.10.017, 19014963

[46] Formisano A Hunsberger M Bammann K Vanaelst B Molnar D Moreno LA . Family structure and childhood obesity: results of the IDEFICS project. Public Health Nutr. (2014) 17:2307–15. doi: 10.1017/S1368980013002474, 24053908 PMC10282634

[47] Hunsberger M Formisano A Reisch LA Bammann K Moreno L de Henauw S . Overweight in singletons compared to children with siblings: the IDEFICS study. Nutr Diabetes. (2012) 2:e35–e. doi: 10.1038/nutd.2012.8 23448718 PMC3408642

[48] Fang K Mu M Liu K He Y. Screen time and childhood overweight/obesity: a systematic review and meta-analysis. Child Care Health Dev. (2019) 45:744–53. doi: 10.1111/cch.12701, 31270831

[49] Tschann JM Martinez SM Penilla C Gregorich SE Pasch LA de Groat CL . Parental feeding practices and child weight status in Mexican American families: a longitudinal analysis. Int J Behav Nutr Phys Act. (2015) 12:66. doi: 10.1186/s12966-015-0224-2, 25986057 PMC4453102

[50] Cai L Lin L Dai M Chen Y Li X Ma J . One-child policy, weight status, lifestyles and parental concerns in Chinese children: a nationwide cross-sectional survey. Eur J Clin Nutr. (2018) 72:1150–8. doi: 10.1038/s41430-018-0178-y, 29748661

[51] Yusoff ZM Shamin F Arif H Adnan NA Nordin NA . School location and mobility effects to obesity cases among primary school children. Adv Sci Lett. (2017) 23:6377–6380. doi: 10.1166/asl.2017.9273

[52] Williams J Scarborough P Townsend N Matthews A Burgoine T Mumtaz L . Associations between food outlets around schools and Bmi among primary students in England: a cross-classified multi-level analysis. PLoS One. (2015) 10:e0132930. doi: 10.1371/journal.pone.0132930, 26186610 PMC4505878

[53] Day PL Pearce J. Obesity-promoting food environments and the spatial clustering of food outlets around schools. Am J Prev Med. (2011) 40:113–21. doi: 10.1016/j.amepre.2010.10.018, 21238858

[54] Hills AP Dengel DR Lubans DR. Supporting public health priorities: recommendations for physical education and physical activity promotion in schools. Prog Cardiovasc Dis. (2015) 57:368–74. doi: 10.1016/j.pcad.2014.09.010, 25269062

[55] Fernandes M Sturm R. Facility provision in elementary schools: correlates with physical education, recess, and obesity. Prev Med. (2010) 50:S30–5. doi: 10.1016/j.ypmed.2009.09.022 19850074 PMC2821448

[56] In-Iw S Saetae T Manaboriboon B. The effectiveness of school-based nutritional education program among obese adolescents: a randomized controlled study. Int J Pediatr. (2012) 2012:608920. doi: 10.1155/2012/608920 23118771 PMC3483824

[57] Jaime PC Lock K. Do school based food and nutrition policies improve diet and reduce obesity? Prev Med. (2009) 48:45–53. doi: 10.1016/j.ypmed.2008.10.018, 19026676

[58] Wang S-F Shu L Sheng J Mu M Wang S Tao X-Y . Birth weight and risk of coronary heart disease in adults: a meta-analysis of prospective cohort studies. J Dev Orig Health Dis. (2014) 5:408–19. doi: 10.1017/s2040174414000440, 25263759

[59] Rossi CE Vasconcelos FDAGD. Birth weight and obesity in children and adolescents: a systematic review. Rev Bras Epidemiol. (2010) 13:246–58. doi: 10.1590/S1415-790X2010000200007

[60] Yu Z Han S Zhu G Zhu C Wang XJ Cao XG . Birth weight and subsequent risk of obesity: a systematic review and meta-analysis. Obes Rev. (2011) 12:525–42. doi: 10.1111/j.1467-789X.2011.00867.x, 21438992

[61] Paliy O Piyathilake CJ Kozyrskyj A Celep G Marotta F Rastmanesh R. Excess body weight during pregnancy and offspring obesity: potential mechanisms. Nutrition. (2014) 30:245–51. doi: 10.1016/j.nut.2013.05.011, 24103493

[62] Mcdonald CM Baylin A Joanne EA Mercedes M-P Eduardo V. Overweight is more prevalent than stunting and is associated with socioeconomic status, maternal obesity, and a snacking dietary pattern in school children from Bogota, Colombia. J Nutr. (2009) 139:370–6. doi: 10.3945/jn.108.098111, 19106320 PMC2646207

[63] Ørskou J Henriksen TB Kesmodel U Secher NJ. Maternal characteristics and lifestyle factors and the risk of delivering high birth weight infants. Obstet Gynecol. (2003) 102:115–20. doi: 10.1097/00006250-200307000-00022, 12850616

[64] Bray GA Bouchard C. The biology of human overfeeding: a systematic review. Obes Rev. (2020) 21:e13040. doi: 10.1111/obr.13040, 32515127

[65] Abbott BD Barber BL. Embodied image: gender differences in functional and aesthetic body image among Australian adolescents. Body Image. (2010) 7:22–31. doi: 10.1016/j.bodyim.2009.10.004, 19945925

[66] Barker ET Galambos NL. Body dissatisfaction of adolescent girls and boys: risk and resource factors. J Early Adolesc. (2003) 23:141–65. doi: 10.1177/0272431603023002002

[67] O'dea JA. School-based health education strategies for the improvement of body image and prevention of eating problems: an overview of safe and successful interventions. Health Educ. (2005) 105:11–33. doi: 10.1108/09654280510572277

[68] Ouyang Y Wang K Zhang T Peng L Song G Luo J. The influence of sports participation on body image, self-efficacy, and self-esteem in college students. Front Psychol. (2020) 10:3039. doi: 10.3389/fpsyg.2019.03039, 32116869 PMC7012809

[69] Hemmingsson E. A new model of the role of psychological and emotional distress in promoting obesity: conceptual review with implications for treatment and prevention. Obes Rev. (2014) 15:769–79. doi: 10.1111/obr.12197, 24931366

[70] Shriver LH Dollar JM Calkins SD Keane SP Shanahan L Wideman L. Emotional eating in adolescence: effects of emotion regulation, weight status and negative body image. Nutrients. (2020) 13:79. doi: 10.3390/nu13010079, 33383717 PMC7824438

[71] Lyter PL. The relationship between the acceptance of the socially constructed ideal body image, body mass index, level of appearance satisfaction and weight management health behaviors in college women. Michigan: The University of Wisconsin-Madison (1997).

[72] Wu HX Ching BH-H He CC Li Y. “Thinness is beauty”: predictors of anti-fat attitudes among young Chinese women. Curr Psychol. (2021) 42:6834–45. doi: 10.1007/s12144-021-02021-x

[73] Pate RR Mitchell JA Byun W Dowda M. Sedentary behaviour in youth. Br J Sports Med. (2011) 45:906–13. doi: 10.1136/bjsports-2011-090192, 21836174

[74] Barnett TA Kelly AS Young DR Perry CK Pratt CA Edwards NM . Sedentary behaviors in today’s youth: approaches to the prevention and management of childhood obesity: a scientific statement from the American Heart Association[J]. Circulation. (2018) 138: e142–e159. doi: 10.1161/CIR.0000000000000591 30354382

[75] Dias PJP Domingos IP Ferreira MG Muraro AP Sichieri R Gonçalves-Silva RMV. Prevalência e fatores associados aos comportamentos sedentários em adolescentes. Rev Saude Publica. (2014) 48:266–74. doi: 10.1590/s0034-8910.2014048004635, 24897048 PMC4206135

[76] Molnar D Schutz Y. The effect of obesity, age, puberty and gender on resting metabolic rate in children and adolescents. Eur J Pediatr. (1997) 156:376–81. doi: 10.1007/s004310050618, 9177980

[77] Tremblay MS Aubert S Barnes JD Saunders TJ Carson V Latimer-Cheung AE . Sedentary behavior research network (SBRN)–terminology consensus project process and outcome. Int J Behav Nutr Phys Act. (2017) 14:1–17. doi: 10.1186/s12966-017-0525-8, 28599680 PMC5466781

[78] Yang J Shen Y Quan X. Physical activity, screen time, and academic burden: a cross-sectional analysis of health among Chinese adolescents. Int J Environ Res Public Health. (2023) 20:4917. doi: 10.3390/ijerph20064917, 36981825 PMC10049325

[79] Zhu X Haegele JA Liu H Yu F. Academic stress, physical activity, sleep, and mental health among Chinese adolescents. Int J Environ Res Public Health. (2021) 18:7257. doi: 10.3390/ijerph18147257, 34299708 PMC8304898

[80] Noor Z Khaliq M Khan AU Ali MA Tahir SK Khaliq K. Academic stress and adolescent health: exploring eating patterns, dietary preferences, and sleep duration in Pakistan's youth. Appetite. (2025) 209:107962. doi: 10.1016/j.appet.2025.107962, 40058607

[81] Hills AP Andersen LB Byrne NM. Physical activity and obesity in children. Br J Sports Med. (2011) 45:866–70. doi: 10.1136/bjsports-2011-090199, 21836171

[82] Vasconcellos F Seabra A Katzmarzyk PT Kraemer-Aguiar LG Bouskela E Farinatti P. Physical activity in overweight and obese adolescents: systematic review of the effects on physical fitness components and cardiovascular associated factors. Sports Med. (2014) 44:1139–52. doi: 10.1007/s40279-014-0193-7, 24743931

[83] Sherry B Jefferds ME Grummer-Strawn LM. Accuracy of adolescent self-report of height and weight in assessing overweight status: a literature review. Arch Pediatr Adolesc Med. (2007) 161:1154–61. doi: 10.1001/archpedi.161.12.1154, 18056560

[84] Zhou X Dibley MJ Cheng Y Ouyang X Yan H. Validity of self-reported weight, height and resultant body mass index in Chinese adolescents and factors associated with errors in self-reports. BMC Public Health. (2010) 10:190. doi: 10.1186/1471-2458-10-190 20384994 PMC2864211

[85] Lin W Shi S Huang H Wen J Chen G. Predicting risk of obesity in overweight adults using interpretable machine learning algorithms. Front Endocrinol (Lausanne). (2023) 14:1292167. doi: 10.3389/fendo.2023.1292167 38047114 PMC10693451

[86] Miguel-Hurtado O Guest R Stevenage SV Neil GJ Black S. Comparing Machine Learning Classifiers and Linear/Logistic Regression to Explore the Relationship between Hand Dimensions and Demographic Characteristics. PLoS One. (2016) 11:e0165521. doi: 10.1371/journal.pone.0165521 27806075 PMC5091918

