# Supplementary material for: Interpretable machine learning for identifying adolescent obesity risk and identifying key determinants
Source: Front Public Health. 2026 Feb 25;14:1657467. doi: 10.3389/fpubh.2026.1657467 (PMC12975944; doi:10.3389/fpubh.2026.1657467)
Supplement: Supplementary file 1 [file Supplementary_file_1.docx]

| Model | Dataset | Accuracy | Sensitivity | Precision | Specificity | AUC |
| --- | --- | --- | --- | --- | --- | --- |
| SVM | Train | 0.8856 | 0.2154 | 0.887 | 0.9955 | 0.8911 |
| SVM | Test | 0.8658 | 0.1442 | 0.5921 | 0.9838 | 0.6765 |
| XGBoost | Train | 0.8864 | 0.2373 | 0.8439 | 0.9928 | 0.8108 |
| XGBoost | Test | 0.8779 | 0.2115 | 0.7253 | 0.9869 | 0.745 |
| GBM | Train | 0.8997 | 0.3224 | 0.9038 | 0.9944 | 0.9062 |
| LightGBM（最优模型） | Test | 0.8788 | 0.2436 | 0.6972 | 0.9827 | 0.7392 |
| Logistic Regression | Train | 0.8685 | 0.0974 | 0.7553 | 0.9948 | 0.7168 |
| Logistic Regression | Test | 0.8712 | 0.1154 | 0.7826 | 0.9948 | 0.7194 |
| Random Forest | Train | 0.8967 | 0.2702 | 0.985 | 0.9993 | 0.8999 |
| Random Forest | Test | 0.877 | 0.1699 | 0.791 | 0.9927 | 0.739 |
| MLP | Train | 0.8723 | 0.1454 | 0.7361 | 0.9915 | 0.7625 |
| MLP | Test | 0.8725 | 0.141 | 0.7458 | 0.9921 | 0.7143 |
