# Supplementary material for: Interpretable machine learning for identifying adolescent obesity risk and identifying key determinants
Source: Front Public Health. 2026 Feb 25;14:1657467. doi: 10.3389/fpubh.2026.1657467 (PMC12975944; doi:10.3389/fpubh.2026.1657467)
Supplement: Supplementary file 2 [file Supplementary_file_2.doc]

A total of 7,397 adolescents were included in this study, comprising 6,356 (85.9%) in the normal weight group and 1,041 (14.1%) in the obese group. Baseline characteristics across individual, family, and school levels were compared between the two groups, with results presented in Table 1.

**Individual-level Variables:**

Statistically significant differences (p < 0.05) were observed between the normal weight and obese groups for most individual-level variables:

Gender: A higher proportion of males was present in the obese group (36.12%) compared to the normal weight group (χ²=71.39, p<0.001).

Birth weight: The obese group included a significantly higher proportion of individuals with high birth weight (>4000 g, 18.06%) and a lower proportion with low birth weight (<2500 g, 9.51%) than the normal weight group (5.16% and 13.85%, respectively; χ²=234.28, p<0.001).

Household registration (Hukou): Adolescents in the obese group were more likely to have non-agricultural (urban) registration (59.85%) compared to those in the normal weight group (46.81%; χ²=60.89, p<0.001).

Body image: The obese group reported significantly more negative body image, with higher proportions of participants rating themselves as “very ugly” (3.17% vs. 1.92%) or “quite ugly” (11.24% vs. 5.05%), and lower proportions as “quite beautiful” (5.96% vs. 8.46%) or “very beautiful” (2.11% vs. 5.65%; χ²=93.40, p<0.001).

Dietary habits: Frequency of consuming unhealthy foods significantly differed between groups (χ²=13.30, p=0.010).

Sleep quality: No significant difference was found between the two groups (p=0.203).

Sedentary time: Median sedentary time was significantly higher in the obese group (6.00 hours) than in the normal weight group (5.00 hours; p<0.001).

Academic workload: The obese group reported a higher academic workload (median=6.00, IQR: 5.00–9.00) compared to the normal weight group (median=6.00, IQR: 5.00–7.00; p<0.001).

Weekend physical activity: No significant difference was observed between groups (p=0.796).

Sports tutoring: Participation in sports tutoring classes did not differ significantly (p=0.911).

Active commuting: A higher proportion of adolescents in the obese group (56.20%) engaged in active commuting compared to the normal weight group (50.69%; χ²=10.85, p<0.001).

Physical activity frequency: The distribution of weekly physical activity sessions differed significantly between groups (χ²=16.08, p=0.024).

**Family-level Variables:**

Paternal and maternal education: Both mother’s and father’s education levels showed significant associations with weight status (p=0.010 for both).

Family economic status: Economic status was a significant factor (χ²=277.95, p<0.001). The proportion of adolescents from “very affluent” families was considerably higher in the obese group (7.20%) than in the normal weight group (0.72%).

Family structure: A significantly higher percentage of obese adolescents (55.04%) were from single-child families compared to the normal weight group (44.29%; χ²=41.68, p<0.001).

**School-level Variables:**

School type: The distribution across school types differed significantly (χ²=12.07, p=0.002), with a higher proportion of obese adolescents in public schools.

School ranking: School ranking was significantly associated with weight status (χ²=238.93, p<0.001). The obese group had a higher proportion of students in “below average” ranking schools (5.48% vs. 0.39%) and a lower proportion in “best” ranking schools (20.46% vs. 28.48%).

School location: The distribution of school locations differed significantly (χ²=100.83, p<0.001). A higher proportion of obese adolescents attended schools in “central urban areas” (57.64% vs. 48.38%) and a lower proportion in “rural areas” (9.22% vs. 21.08%).

Sports facilities: A small but significant difference was found in sports facilities availability (p=0.045), with the obese group showing a slightly higher median score.

Table 1 Comparison of baseline data between normal and obese groups

|  | **Variables** | **Total(n=7397)** | **Normal(n=6356)** | **Obesity(n=1041)** | **statistic** | ***P*** |
| --- | --- | --- | --- | --- | --- | --- |
| **Individual-level variables** | **Gender, n(%)** |  |  |  | χ²=71.39 | **<.001** |
|  | Male | 3569 (48.25) | 3193 (50.24) | 376 (36.12) |  |  |
|  | Female | 3828 (51.75) | 3163 (49.76) | 665 (63.88) |  |  |
|  | **Birth Weight, n(%)** |  |  |  | χ²=234.28 | **<.001** |
|  | Light | 979 (13.24) | 880 (13.85) | 99 (9.51) |  |  |
|  | Normal | 5902 (79.79) | 5148 (80.99) | 754 (72.43) |  |  |
|  | Heavy | 516 (6.98) | 328 (5.16) | 188 (18.06) |  |  |
|  | **Household Registration, n(%)** |  |  |  | χ²=60.89 | **<.001** |
|  | Rural | 3799 (51.36) | 3381 (53.19) | 418 (40.15) |  |  |
|  | Urban | 3598 (48.64) | 2975 (46.81) | 623 (59.85) |  |  |
|  | **Body Image, n(%)** |  |  |  | χ²=93.40 | **<.001** |
|  | Very ugly | 155 (2.10) | 122 (1.92) | 33 (3.17) |  |  |
|  | Quite ugly | 438 (5.92) | 321 (5.05) | 117 (11.24) |  |  |
|  | Average | 5823 (78.72) | 5016 (78.92) | 807 (77.52) |  |  |
|  | Quite beautiful | 600 (8.11) | 538 (8.46) | 62 (5.96) |  |  |
|  | Very beautiful | 381 (5.15) | 359 (5.65) | 22 (2.11) |  |  |
|  | **Dietary Habits, n(%)** |  |  |  | χ²=13.30 | **0.010** |
|  | Never | 328 (4.43) | 274 (4.31) | 54 (5.19) |  |  |
|  | Rarely | 2637 (35.65) | 2288 (36.00) | 349 (33.53) |  |  |
|  | Sometimes | 3346 (45.23) | 2836 (44.62) | 510 (48.99) |  |  |
|  | Often | 970 (13.11) | 851 (13.39) | 119 (11.43) |  |  |
|  | Always | 116 (1.57) | 107 (1.68) | 9 (0.86) |  |  |
|  | **Sleep Quality, M (Q₁, Q₃)** | 0.00 (0.00, 1.00) | 0.00 (0.00, 1.00) | 0.00 (0.00, 1.00) | Z=-1.27 | 0.203 |
|  | **Sedentary Time, M (Q₁, Q₃)** | 5.00 (4.00, 6.00) | 5.00 (4.00, 6.00) | 6.00 (4.00, 7.00) | Z=-7.64 | **<.001** |
|  | **Academic Workload, M (Q₁, Q₃)** | 6.00 (5.00, 8.00) | 6.00 (5.00, 7.00) | 6.00 (5.00, 9.00) | Z=-7.68 | **<.001** |
|  | **Weekend Physical Activity, n(%)** |  |  |  | χ²=0.07 | 0.796 |
|  | No | 2691 (36.38) | 2316 (36.44) | 375 (36.02) |  |  |
|  | Yes | 4706 (63.62) | 4040 (63.56) | 666 (63.98) |  |  |
|  | **Sports Tutor, n (%)** |  |  |  | χ²=0.01 | 0.911 |
|  | No | 6849 (92.59) | 5886 (92.61) | 963 (92.51) |  |  |
|  | Yes | 548 (7.41) | 470 (7.39) | 78 (7.49) |  |  |
|  | **Active Commute, n(%)** |  |  |  | χ²=10.85 | **<.001** |
|  | No | 3590 (48.53) | 3134 (49.31) | 456 (43.80) |  |  |
|  | Yes | 3807 (51.47) | 3222 (50.69) | 585 (56.20) |  |  |
|  | **PA Freq, n(%)** |  |  |  | χ²=16.08 | **0.024** |
|  | Never | 209 (2.83) | 164 (2.58) | 45 (4.32) |  |  |
|  | once | 1046 (14.14) | 913 (14.36) | 133 (12.78) |  |  |
|  | Twice | 1822 (24.63) | 1571 (24.72) | 251 (24.11) |  |  |
|  | Three times | 1140 (15.41) | 962 (15.14) | 178 (17.10) |  |  |
|  | Four times | 469 (6.34) | 412 (6.48) | 57 (5.48) |  |  |
|  | Five times | 1733 (23.43) | 1500 (23.60) | 233 (22.38) |  |  |
|  | Six times | 232 (3.14) | 196 (3.08) | 36 (3.46) |  |  |
|  | Seven times | 746 (10.09) | 638 (10.04) | 108 (10.37) |  |  |
| **Family-level variables** | **M Edu, n (%)** |  |  |  | χ²=15.02 | **0.010** |
|  | No formal education | 0 (0) | 0 (0) | 0 (0) |  |  |
|  | Primary school | 1416 (19.14) | 1242 (19.54) | 174 (16.71) |  |  |
|  | Junior secondary school | 3122 (42.21) | 2701 (42.50) | 421 (40.44) |  |  |
|  | Technical secondary school / Vocational school / Vocational high school / Regular high school | 1774 (23.98) | 1514 (23.82) | 260 (24.98) |  |  |
|  | Associate degree | 456 (6.16) | 374 (5.88) | 82 (7.88) |  |  |
|  | Bachelor's degree | 558 (7.54) | 463 (7.28) | 95 (9.13) |  |  |
|  | Master's degree or above | 71 (0.96) | 62 (0.98) | 9 (0.86) |  |  |
|  | **F Edu, n (%)** |  |  |  | χ²=15.18 | **0.010** |
|  | No formal education | 0 (0) | 0 (0) | 0 (0) |  |  |
|  | Primary school | 867 (11.72) | 749 (11.78) | 118 (11.34) |  |  |
|  | Junior secondary school | 3160 (42.72) | 2760 (43.42) | 400 (38.42) |  |  |
|  | Technical secondary school / Vocational school / Vocational high school / Regular high school | 2082 (28.15) | 1777 (27.96) | 305 (29.30) |  |  |
|  | Associate degree | 482 (6.52) | 396 (6.23) | 86 (8.26) |  |  |
|  | Bachelor's degree | 693 (9.37) | 580 (9.13) | 113 (10.85) |  |  |
|  | Master's degree or above | 113 (1.53) | 94 (1.48) | 19 (1.83) |  |  |
|  | **Fam Econ, n(%)** |  |  |  | χ²=277.95 | **<.001** |
|  | Very difficult | 176 (2.38) | 155 (2.44) | 21 (2.02) |  |  |
|  | Relatively difficult | 818 (11.06) | 766 (12.05) | 52 (5.00) |  |  |
|  | Moderate | 5446 (73.62) | 4697 (73.90) | 749 (71.95) |  |  |
|  | Relatively affluent | 836 (11.30) | 692 (10.89) | 144 (13.83) |  |  |
|  | Very affluent | 121 (1.64) | 46 (0.72) | 75 (7.20) |  |  |
|  | **Fam Struct, n(%)** |  |  |  | χ²=41.68 | **<.001** |
|  | No | 3388 (45.80) | 2815 (44.29) | 573 (55.04) |  |  |
|  | Yes | 4009 (54.20) | 3541 (55.71) | 468 (44.96) |  |  |
| **School-level variables** | **School Type, n(%)** |  |  |  | χ²=12.07 | **0.002** |
|  | Public schools | 6942 (93.85) | 5948 (93.58) | 994 (95.49) |  |  |
|  | Private schools | 356 (4.81) | 327 (5.14) | 29 (2.79) |  |  |
|  | Private schools for children of migrant workers | 99 (1.34) | 81 (1.27) | 18 (1.73) |  |  |
|  | **School Rank, n(%)** |  |  |  | χ²=238.93 | **<.001** |
|  | Worst | 484 (6.54) | 420 (6.61) | 64 (6.15) |  |  |
|  | Below Average | 82 (1.11) | 25 (0.39) | 57 (5.48) |  |  |
|  | Middle | 974 (13.17) | 804 (12.65) | 170 (16.33) |  |  |
|  | Above Average | 3834 (51.83) | 3297 (51.87) | 537 (51.59) |  |  |
|  | Best | 2023 (27.35) | 1810 (28.48) | 213 (20.46) |  |  |
|  | **School Loc, n(%)** |  |  |  | χ²=100.83 | **<.001** |
|  | Rural areas | 1436 (19.41) | 1340 (21.08) | 96 (9.22) |  |  |
|  | Townships | 987 (13.34) | 873 (13.74) | 114 (10.95) |  |  |
|  | Urban-rural Fringe areas | 908 (12.28) | 751 (11.82) | 157 (15.08) |  |  |
|  | Peripheral urban areas | 391 (5.29) | 317 (4.99) | 74 (7.11) |  |  |
|  | Central urban areas | 3675 (49.68) | 3075 (48.38) | 600 (57.64) |  |  |
|  | Sports Fac, n(%) |  |  |  | χ²=30.72 | **<.001** |
|  | 3 | 182 (2.46) | 138 (2.17) | 44 (4.23) |  |  |
|  | 4 | 5451 (73.69) | 4733 (74.47) | 718 (68.97) |  |  |
|  | 5 | 1567 (21.18) | 1308 (20.58) | 259 (24.88) |  |  |
|  | 6 | 197 (2.66) | 177 (2.78) | 20 (1.92) |  |  |

Z: Mann-Whitney test, χ²: Chi-square test. M: Median, Q₁: 1st Quartile, Q₃: 3rd Quartile
