# Supplementary material for: Interpretable machine learning for identifying adolescent obesity risk and identifying key determinants
Source: Front Public Health. 2026 Feb 25;14:1657467. doi: 10.3389/fpubh.2026.1657467 (PMC12975944; doi:10.3389/fpubh.2026.1657467)
Supplement: Supplementary file 3 [file Supplementary_file_3.doc]

**1a**


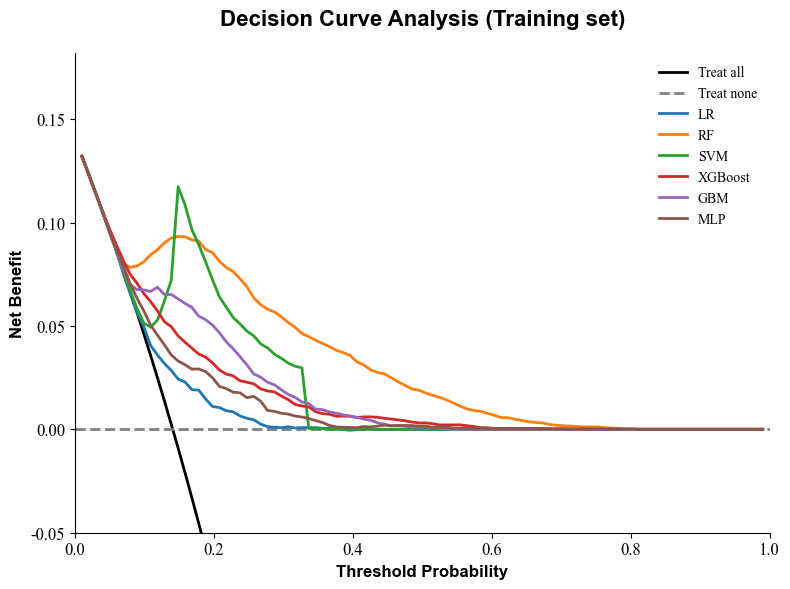


**1b**


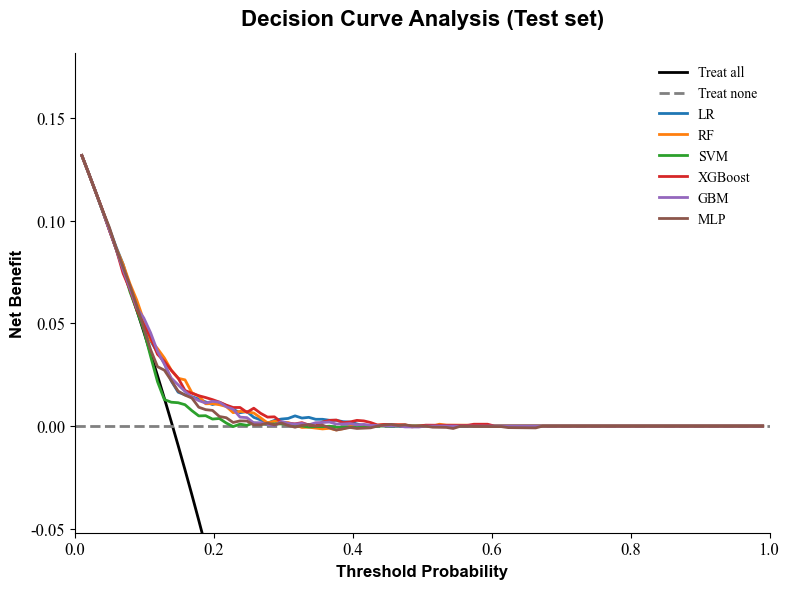


Figure 1 Decision Curve Analysis for the Machine Learning Models in the (4a) Training and (4b) Test Sets
